# Supplementary material for: A molecular standard for circulating HBV RNA detection and quantification assays in patients with chronic hepatitis B
Source: JHEP Rep. 2024 May 25;6(10):101124. doi: 10.1016/j.jhepr.2024.101124 (PMC11424956; doi:10.1016/j.jhepr.2024.101124)
Supplement: Multimedia component 1 [file mmc1.pdf]

A molecular standard for circulating HBV RNA detection and quantification assays in chronic hepatitis B patients

Alexia Paturel, Francesca Casuscelli di Tocco, Delphine Bousquet, Marie-Laure Plissonnier, Xavier Grand, Hyosun Tak, Françoise Berby, Caroline Scholtès, Barbara Testoni, Fabien Zoulim, Massimo Levrero

Table of contents

Fig. S1 2

Fig. S2 3

Fig. S3 4

Fig. S4 5

Fig. S5 6

Fig. S6 7

Figure S1

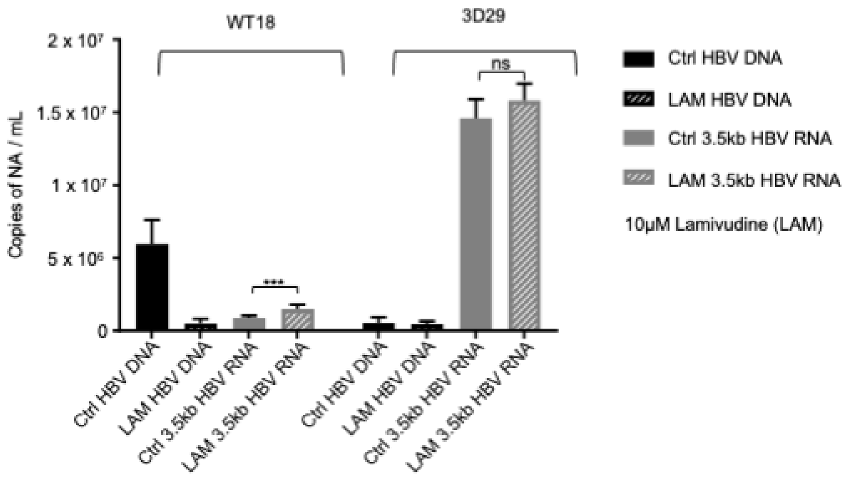

Fig. S1 – Effect of lamivudine treatment on viral nucleic acids secretion from the Huh7-WT18 and Huh7-3D29 cell clones. ddPCR quantification of HBV DNA and RNA secreted from Huh7-WT18 and Huh7-3D29 clonal cell lines untreated (Ctrl) or treated with 10µM of lamivudine (LAM). Huh7-WT18 control HBV RNA copies/mL in the supernatant vs Huh7-WT18 LAM treated cells (Paired t-test; P = 0.0001). Huh7-3D29 control HBV RNA copies/mL in the supernatant vs Huh7-3D29 LAM treated cells (Paired t-test; P = 0.3255). Validation of samples distribution as parametric by Shapiro-Wilk test. (Shapiro-Wilk p-value = 0.00062610). Error bars represent standard deviation of 2 technical replicates from each of 3 distinct cell passages, which served as biological replicates.

Figure S2

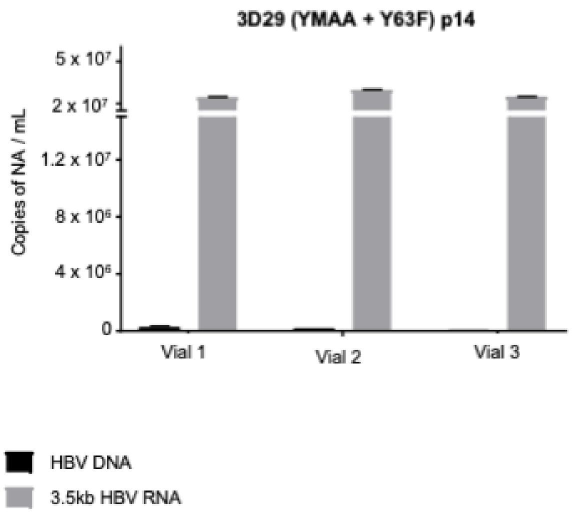

Fig. S2 - The Huh7-3D29 clone secretory phenotype is consistant between vials issued of the same culture passage. ddPCR quantification of secreted HBV DNA and RNA from Huh7-3D29 clone in different vials from passage 14 (one-way Kruskal-Wallis test, P = 0.0667).

Figure S3

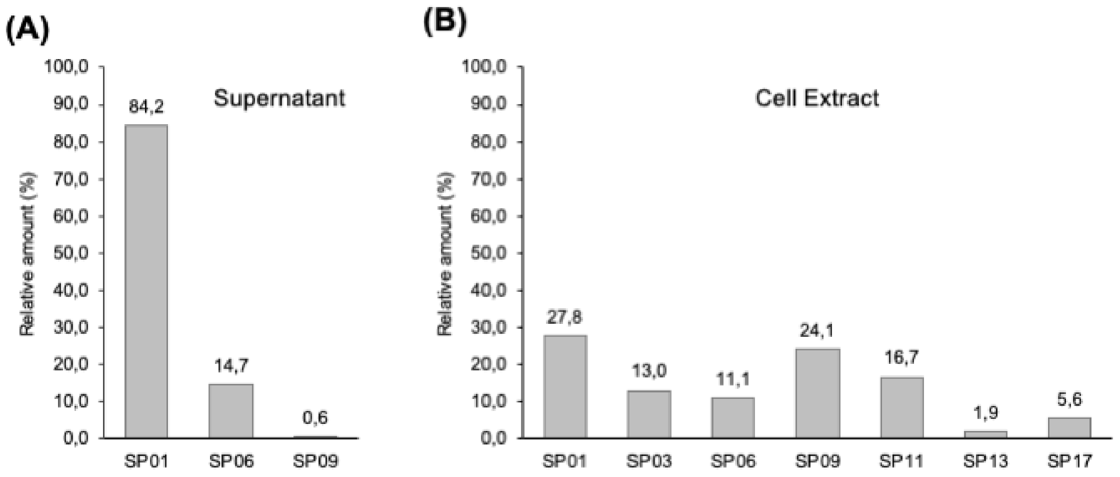

Fig. S3 - Percentage of each splice variant species of the 3.5kb RNA transcript. Values obtained from Nanopore sequencing performed on amplification products in the cell culture supernatants (*left panel*) and cellular extract of Huh7-3D29 cells (*right panel*) obtained after 5'RACE and calculated from transcripts identified as 3.5 according to TSS. All spliced variants below 0,3% was not included in the graph. SPs = splice variants.

Figure S4

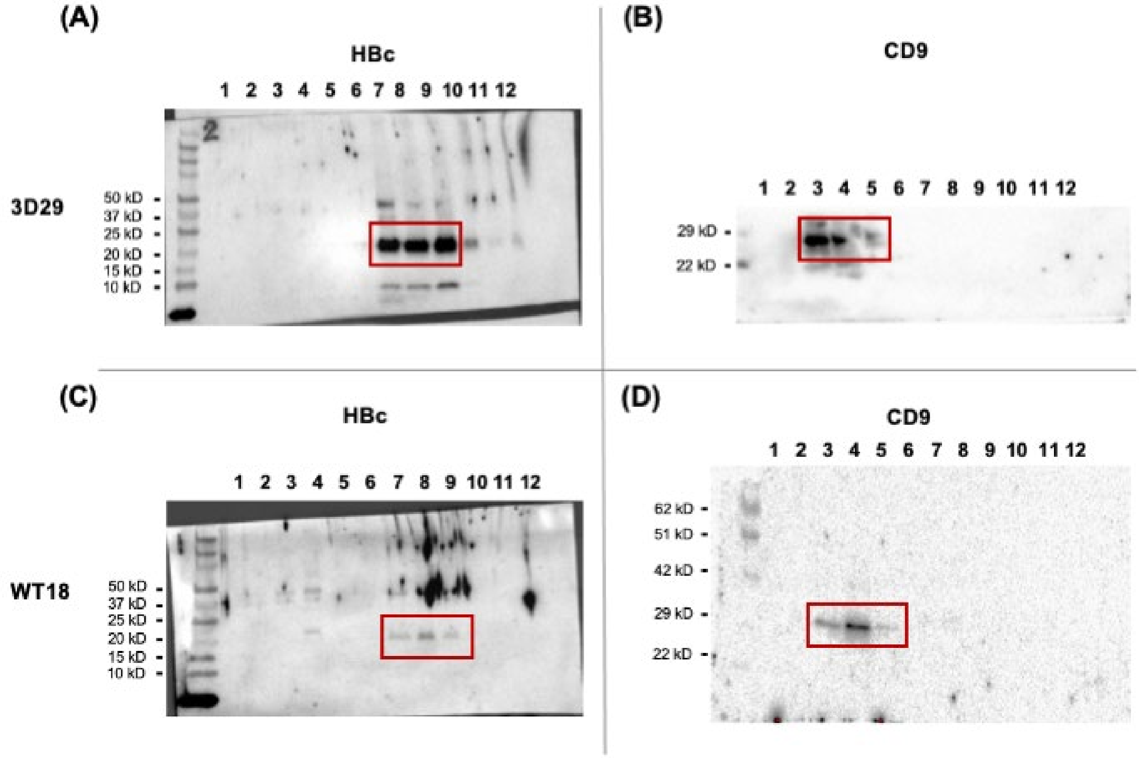

Figure S4 – Western Blot Raw data

Fig. S4 – HBc and CD9 immunoblot raw data. The entire immunoblots for the HBV capsid (HBc) and the sEV CD9 protein used to populate Figure 3 are shown. The figures on the top represents the 12 fractions obtained from the sucrose / iodixanol gradients of Huh7-3D29 (panels A and B) and Huh7-WT18 (panels C and D) cell supernatants. For the anti-HBc immunoblots (panels A and C) the Precision Plus Protein™ Prestained Protein Standard- (Biorad) was used. For the anti-CD9 immunoblots (panels B and D) the Opti-protein Marker (Applied Biological Material) and a Tris-Glycine 15% gel were used. In (B) the upper part of immunoblot membrane was cutted out in order to reduce the background noise and allow a longer exposure time.

Figure S5

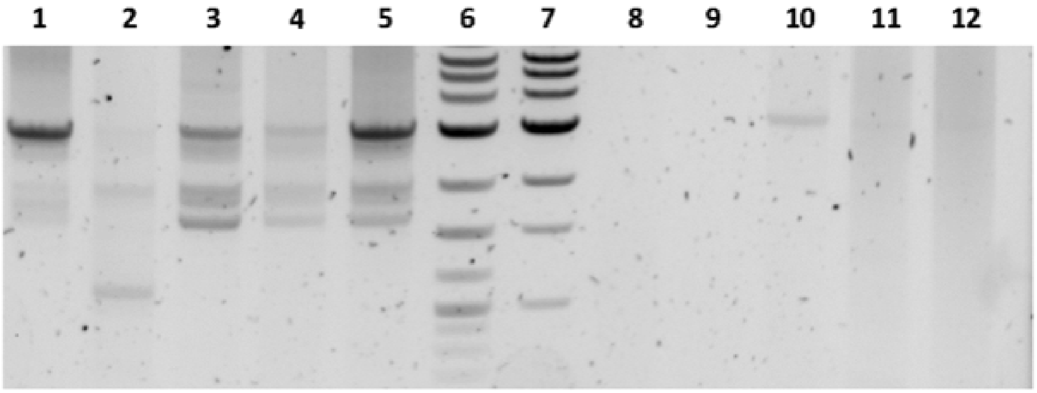

Fig. S5. PAGE analysis of the 5'RACE performed on cell extracts and culture supernatants. Lanes 3, 6 and 10 are included in the main figures as Figure 4A. Lanes 1-5: intracellular fractions (1 = HepAD38; 2 = PLC/PRF/5; 3 = Huh7-3D29; 4 = Huh7-5D1; 5 = Huh7-WT18). Lanes 6-7: molecular weight (MW) markers. Lanes 7-12: cell culture supernatants (8 = HepAD38; 9 = PLC/PRF/5; 10 = Huh7-3D29; 11 = Huh7-5D1; 12 = Huh7-WT18

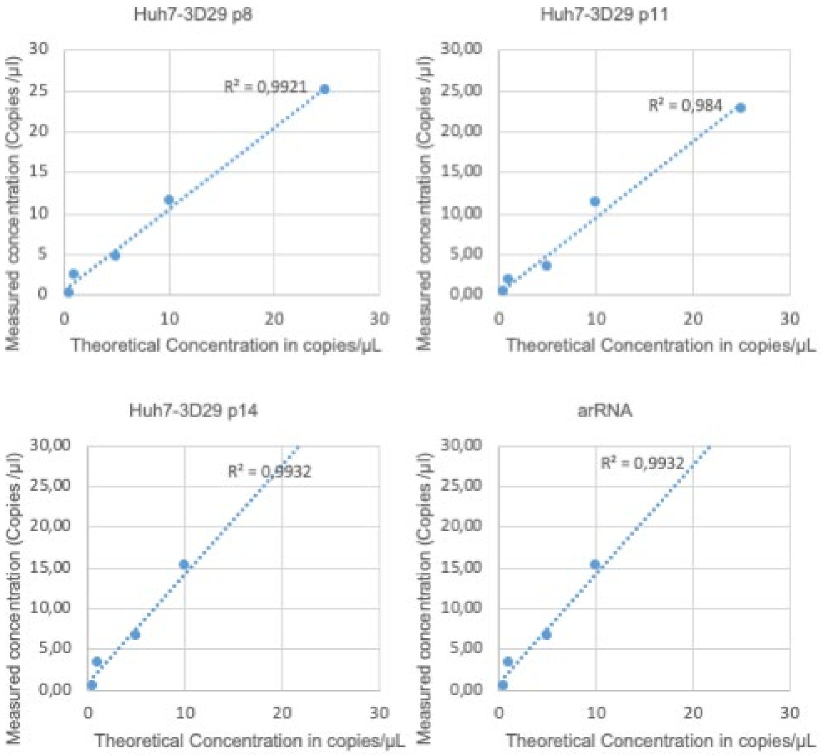

Fig. S6 – Performance of low concentration serial dilutions of Huh7-3D29 cell culture supernatant as a standard for HBV RNAs quantification assays. Serial dilutions from 25 until 0,5 copies/µl of Huh7-3D29 cell supernatants from 3 passages and of the HBV arRNA were used for the 2-step ddPCR. R2 = Spearman's coefficient correlation.
