# Supplementary material for: A molecular standard for circulating HBV RNA detection and quantification assays in patients with chronic hepatitis B
Source: JHEP Rep. 2024 May 25;6(10):101124. doi: 10.1016/j.jhepr.2024.101124 (PMC11424956; doi:10.1016/j.jhepr.2024.101124)
Supplement: Multimedia component 2 [file mmc2.pdf]

JHEP Reports

CTAT methods

Tables for a "Complete, Transparent, Accurate and Timely account" (CTAT) are now mandatory for all revised submissions. The aim is to enhance the reproducibility of methods.

- Only include the parts relevant to your study
- Refer to the CTAT in the main text as 'Supplementary CTAT Table'
- Do not add subheadings
- Add as many rows as needed to include all information
- Only include one item per row

If the CTAT form is not relevant to your study, please outline the reasons why:

1.1 Antibodies

Name	Citation	Supplier	Cat no.	Clone no.
CD9		Thermo fisher Scientific	cat.10626D	
HBc		Abcam	cat. ab115992	

1.2 Cell lines

Name	Citation	Supplier	Cat no.	Passage no.	Authentication test method
Huh7 cell line	Nakabayashi et al. 1982 (2) Durantel et al. (1)	Hepatoma Huh7 cell line was a kind gift from Christoph Seeger and is described in Durantel et al. (1)		P9	Order ID:11107457633 Eurofins, CLA service

1.3 Sequence based reagents

Name	Sequence	Supplier
3' HBV specific Gsp1 primer	5'-TTAGGCAGAGGTGAAAAAAGTTG-3'	Eurogentec
GENERACER primer	5-CGACTGGAGCACGAGGACACTGA-3'	Eurogentec
GeneRacer Adaptor	5'CGACUGGAGCACGAGGACACUGACAUGGACUGAAGGAGUAGAAA-3'	Eurogentec
3.5kb HBV RNA Forward primer	5'-ggagtgtggattcgcactcct-3'	Eurogentec
3.5kb HBV RNA Reverse primer	5'-agattgagatcttctgcgac-3'	Eurogentec
3.5kb HBV RNA probe	5'aggcaggtcccctagaagaagaactcc-3'	Tibmolbiol

HBV Taqman probe/primers solution	Pa03453406_s1	Thermofischer

1.4 Deposited data

Name of repository	Identifier	Link
Collection Nationale de Cultures de Microorganismes (CNCM)	Huh7 – HBV 3D29	CNCM I-5878
Collection Nationale de Cultures de Microorganismes (CNCM)	Huh7 – HBV WT18	CNCM I-5879

1.5 Software

Software name	Manufacturer	Version
GraphPad Prism		v7.05
guppy	Oxford Nanopore Technologies	6.4.6
pycoQC		2.5.2
porechop		0.2.4
seqkit		2.1.0
cutadapt		3.5
minimap2		2.21-r1071
samtools		1.7
nanosplicer		1.0
R software		4.1.2

1.6 Other (*e.g.* drugs, proteins, vectors etc.)

DMEM (Dulbecco's Modified Eagle Medium)	Thermo Fisher scientific (Gibco)	11960044
GlutaMAX™ Supplement	Thermo Fisher scientific (Gibco)	35050038
MEM Non-Essential Amino Acids Solution	Thermo Fisher scientific (Gibco)	11140035
Sodium Pyruvate (100 mM)	Thermo Fisher scientific (Gibco)	11360070
Penicillin Streptomycin	Thermo Fisher scientific (Gibco)	15140122
trypsin-EDTA	Thermo Fisher scientific (Gibco)	25300-054
Blasticidin	InvivoGen	Ant-bl-1

pTriEX-Bsd vector	Kindly provided from David Durantel. Described Durantel et al. in (1)	
Mirus Bio™ TransIT™-2020 Reagent	Fisher scientific	MIR5400
Opti-MEM medium	Thermo Fisher scientific	31985062
High Pure Viral Nucleic Acid	(Roche, Diagnostics)	11858874001
RQ1 RNase-Free DNase	Promega	Cat#M6101
SuperScript^{TM} IV VILO^{TM} Master Mix	Invitrogen	Cat # 11766500
2X ddPCR Supermix™ for probes (no dUTP)	BioRad	1863025
RNA 5' Pyrophosphohydrolase (RppH)	New England Biolabs	M0356S
SuperScript reverse transcriptase IV	ThermoFisher Scientific	18090050
Prime Star super mix DNA Polymerase	TAKARA	R047A
Rapid sequencing gDNA kit	Oxford Nanopore	SQK-PBK004
OptiPrep Density Gradient Medium	sigma-Aldrich	D1556
Lamivudine	sigma-Aldrich	L1295

1.7 Please provide the details of the corresponding methods author for the manuscript:

Prof. Massimo Levrero
Institute of Hepatology Lyon (IHL)
Cancer Research Center of Lyon (CRCL) - INSERM U1052
151 cours Albert Thomas
69424 Lyon Cedex 03
E-mail: massimo.levrero@inserm.fr

2.0 Please confirm for randomised controlled trials all versions of the clinical protocol are included in the submission. These will be published online as supplementary information.

NA

1. Durantel D, et al. A new strategy for studying in vitro the drug susceptibility of clinical isolates of human hepatitis B virus. Hepatology. 2004;40:855–864. doi: 10.1002/hep.20388.
2. Nakabayashi H, Taketa K, Miyano K, Yamane T, Sato J. Growth of human hepatoma cells lines with differentiated functions in chemically defined medium. Cancer Res. 1982 Sep;42(9):3858-63. PMID: 6286115.
