# Supplementary material for: A molecular standard for circulating HBV RNA detection and quantification assays in patients with chronic hepatitis B
Source: JHEP Rep. 2024 May 25;6(10):101124. doi: 10.1016/j.jhepr.2024.101124 (PMC11424956; doi:10.1016/j.jhepr.2024.101124)
Supplement: Multimedia component 4 [file mmc4.pdf]

A molecular standard for circulating HBV RNA detection and quantification assays in patients with chronic hepatitis B

Alexia Paturel[1,2,3], Francesca Casuscelli di Tocco[1,2,3], Delphine Bousquet[1,2,3], Marie-Laure Plissonnier[1,2], Xavier Grand[1,2,3], Hyosun Tak[1,2,3], Françoise Berby[1,4], Caroline Scholtès[1,2,3,5], Barbara Testoni[1,2], Fabien Zoulim[1,2,3,4], Massimo Levrero[1,2,3,4,6,*]

JHEP Reports **2024**. vol. 6 | 1–11

Check for updates

Background & Aims: Circulating HBV RNAs have been proposed as a biomarker that reflects the transcriptional activity of covalently closed circular DNA (cccDNA) and may help to evaluate HBV treatment activity. Different research assays have been proposed and, although two PCR-based research use only investigational assays have been developed, the lack of standardized protocols represents an important limitation. Here we have designed and generated a stable clonal cell line producing an RNA-based standard for the calibration of PCR-based circulating HBV RNA assays.

Methods: HBV RNA-producing Huh7-derived stable cell lines were generated by transfecting pTriEX plasmids containing 1.1 unit length HBV DNA genomes carrying mutations in the catalytic site (YMAA mutation) and the TP domain (Y63F) of the polymerase, and the ε-loop of the pregenomic (pg)RNA (mutation A1G).

Results: The clonal cell line (Huh7-3D29), carrying a double YMAA and Y63F mutation, displayed, and maintained over several passages in culture, a high RNA secretion phenotype with negligible residual secreted HBV DNA. Density gradient centrifugation showed that most of the secreted HBV RNA from Huh7-3D29 cells was detected in naked capsid and virion-like particles and only a minority in small extracellular vescicles. Nanopore sequencing of 5'RACE products shows that the majority of the Huh7-3D29-secreted HBV RNAs start at the 5' end of pgRNA and pgRNA-derived spliced RNAs. Finally, Huh7-3D29 cells showed a high and up-scalable secreted RNA yield allowing 1,300 standard curves in 9 days from one flask.

Conclusion: We generated a clonal cell line that produces high quantities of HBV RNAs with very low quantities of contaminating HBV DNAs, representing a stable source of RNA standard for HBV RNA assay calibration.

© 2024 The Authors. Published by Elsevier B.V. on behalf of European Association for the Study of the Liver (EASL). This is an open access article under the CC BY license (http://creativecommons.org/licenses/by/4.0/).

Introduction

HBV remains a major public health problem worldwide, despite the availability of an effective vaccine and antiviral therapies. 296 million people were living with chronic hepatitis B (CHB) infection in 2019, with 0.5-1 million dying annually because of HBV-related advanced liver disease.[1] Long-term treatment with anti-HBV nucleos(t)side analogues (NUCs) suppresses viral replication, liver inflammation and disease progression.[2] However, only a small proportion of patients achieve a *functional HBV cure*, defined by a sustained off-treatment loss of hepatitis B surface antigen (HBsAg) that is associated with an improved long-term clinical outcome and the ability to stop treatment with very low rates of disease reactivation.[3,4] Innate and adaptive antiviral immune responses control the residual covalently closed circular DNA (cccDNA) reservoir.[5] A *complete HBV cure* with eradication of all cccDNA in the liver and no residual risk of viral reactivation, is rarely, if ever, achieved with NUCs. The development of new antiviral therapies targeting the cccDNA reservoir is a major move towards *HBV cure*.[5] Several new molecular entities (NMEs) with direct antiviral or immunomodulatory activities are being developed, with the aim of inducing cure with a finite treatment duration.

Direct assessment of cccDNA levels and activity requires liver biopsy and cccDNA quantification remains technically challenging.[6–8] To assist the clinical development of these NMEs, there is an urgent need for the development of non-invasive biomarkers that adequately reflect the size and transcriptional activity of intrahepatic cccDNA. HBV DNA and HBsAg, the currently used biomarkers to define HBV endpoints have several limitations in this setting: i) serum HBV DNA is the best indicator of the antiviral activity of a new treatment in naive patients but it is already suppressed in NUC-treated patients; ii) HBsAg loss can at the same time act as a marker of target engagement, reflecting the direct activity of specific NMEs and the endpoint of all new therapies; iii) the predictive value of different thresholds of HBsAg decrease remains to be validated and the kinetics of HBsAg decrease are often too slow to allow for early outcome prediction in clinical trials. In addition, HBsAg may be expressed from the viral genome integrated into the host genome and thus its level may not fully reflect the

* Corresponding author. Address: Institute of Hepatology Lyon (IHL), Cancer Research Center of Lyon (CRCL) - INSERM U1052, 151 cours Albert Thomas, 69424 Lyon Cedex 03, France.
E-mail address: massimo.levrero@inserm.fr (M. Levrero).
https://doi.org/10.1016/j.jhepr.2024.101124

transcriptional activity of cccDNA.[9,10] Thus, new biomarkers would be useful: a) to better characterize patients in the different phases of HBV infection and disease; b) to predict response to interferon (IFN) at an early stage, so that treatment is only extended in patients with a high probability of achieving a functional cure; c) to identify patients that could discontinue NUCs without risk of relapse; d) to assess target engagement and antiviral activity in patients receiving NMEs; e) to predict HBsAg response, *i.e.* functional cure, in patients receiving NMEs.[7,11]

Several studies have identified circulating HBV RNAs as a biomarker reflecting the transcriptional activity of cccDNA[12,13] and have shown that circulating HBV RNA may help monitor CHB infection.[11,14,15] Although the potential relevance of circulating HBV RNAs as a biomarker is well established, a major limitation is the lack of standardized protocols (*e.g.*, sample extraction, reverse transcription, choice of primers for amplification, genotype inclusivity, specificity for RNA). Investigational research use only (RUO) assays to quantify circulating HBV RNAs have been developed by industry.[16–18] Different standards have been used for the two RUOs: a) the World Health Organization (WHO) International Standard (IS) for HBV DNA,[16] originally established to be used as a calibrator for HBV DNA nucleic acid amplification technique-based assays;[19–24] b) a synthetic armored RNA (arRNA) derived from the 3' end of HBV pregenomic RNA (pgRNA).[16–18] The appropriateness of the WHO HBV DNA standard has been challenged.[25] Indeed, it is questionable whether an HBV RNA unit is equivalent to an international HBV DNA unit defined by the plasma derived WHO HBV DNA standard that also contains circulating HBV RNA.[25] Conversely, arRNA synthesis is unpractical and costly for routine use. The lack of a unique, recognized calibrator for circulating HBV RNA quantification remains a potentially significant limitation to compare results generated by different assays.

Herein, we describe the generation of a stable clonal cell line producing an RNA-based standard for the calibration of circulating HBV RNA assays that are used to evaluate the size of the transcriptionally active cccDNA pool in a non-invasive manner and the activity of novel strategies aimed at curing HBV infection.

Materials and methods

Cell lines

The human Huh7 cell line was cultured at 37 °C in a humidified atmosphere containing 5% CO_2 in Dulbecco's modified Eagle medium supplemented with 10% Hyclone fetal clone II serum 1% GlutaMax (Gibco), 1% Penicillin/Streptomycin (Gibco), 1% sodium pyruvate (Gibco), 1% non-essential amino acids (NEAA, Gibco).

Plasmids

The HBV RNA-producing cell lines was generated by transfecting a pTriEX plasmid derivative containing 1.1-unit length WT and mutated HBV DNA genomes in Huh7 cells. In total, three mutations were selected: YMAA and Y63F in the polymerase open reading frame, and A1G in the ε-loop region. Five HBV mutated genomes and a wild-type (WT) HBV genome of genotype D have been generated and inserted into the pTriEX-Bsd vector: a plasmid containing the WT 1.1 unit HBV genome, two plasmids containing the 1.1 unit HBV genome carrying the YMAA mutation or the Y63F mutation and three plasmids containing the 1.1 unit HBV genome carrying the following combinations of mutations: YMAA + Y63F, A1G + YMAA, and A1G + Y63F. All plasmids include the blasticidin resistance gene for cell culture selection. WT and mutated 1.1-unit HBV genomes were synthetized and the plasmids produced and controlled by sequencing by GenScript®.

Cell transfection and culture

Huh7 cells were seeded at 60–80% confluency and were allowed to adhere overnight. Cells were then transfected with the indicated amounts of total plasmid DNA with the TransIT®-2020 kit (Mirus) in serum-free Opti-MEM medium (Life Technologies), following the manufacturer's instructions. After 12h, the transfection medium was removed and was replaced with fresh medium. Transfected cells were grown and amplified in selective growth media (Huh7 medium described above) supplemented with 4 μg/ml of Blasticidin.

Clonal cell line isolation and expansion

Colony formation was obtained after serial dilution of Huh7 polyclonal cell population from 1×10^4 cells to 100 cells per 150 cm^2 petri dish. Cell plates were incubated at 37 °C with 5% CO_2 for 20 days to allow colony formation and growth. Using single channel pipettor, single colonies were transferred to 1 ml PBS. Cells were pelleted by centrifugation for 5 min at 900 rpm and resuspended in 50 μl of 0.25% trypsin-EDTA (Gibco) and kept for 5 min at 37 °C. Cells were then plated in 24 wells plates with 1 ml fresh Huh7 media. Clonal cell lines were amplified under blasticidin selection. Clones to be further carried on were chosen based on cell growth, HBV RNA/HBV DNA ratio, and HBV RNA productivity.

High scale supernatant production

To test the stability over time of the HBV RNA secretory phenotype and to perform higher scale supernatant collection to produce the HBV RNA standard, cells were seeded (25×10^6 cells/175 cm^2 flask) and cultured overnight before change of media containing 2.5% DMSO. To collect large quantities of HBV RNAs, cells were kept under standard culture conditions for 9 days. Every 3 days, supernatant was collected, and fresh media was added. After 9 days, all collected supernatants were pooled and centrifuged for 5 min at 1,500 rpm to remove any cellular debris.

Ultracentrifugation, DNA and RNA isolation

Pooled cell culture supernatants were ultracentrifuged for 5 h at 25,000 rpm over a 4 ml 20% w/v sucrose cushion. After ultracentrifugation, the supernatant was removed, and the virus-containing pellet was resuspended in 600 μl of PBS 1X. 28.5 μl aliquots (28.5 μl out of 600 μl) of each resuspended pellet was completed to 200 μl with PBS 1X. DNA and RNA from resuspended pellets were extracted using High Pure Viral Nucleic Acid (Roche, Diagnostics) according to the manufacturers' instructions. The same final volume of extracted nucleic acid elution (40 μl) was used for DNA or RNA analysis. RNA samples

were treated with RQ1 RNase-Free DNase (Promega, Cat# M6101) for 30 min at 37 °C and stored until use.

HBV nucleic acids quantification by 2-step droplet digital PCR (ddPCR)

For total HBV DNA analysis 4 µl of eluted DNA were used. For HBV RNA analysis, 4 µl of DNase-treated RNA was reverse transcribed and amplified using the SuperScript™ IV VILO™ Master Mix (Invitrogen, Cat #11766500). The 22 µl amplification mixtures comprise 11 µl of 2X ddPCR Supermix™ for probes (no dUTP) (Bio-Rad), 1.1 µl of primers and probe mix, and 5 µl of either DNA or cDNA. Nucleic acid inputs were adjusted to have acceptable rates of *negative events*: samples were diluted 1:5. Probes and primers include: the HBV Pa03453406_s1 primers and probe set (Thermofischer) for both total HBV DNA and total HBV RNA quantification and the forward primer ggagtgtggattcgcactcct, reverse primer agattgagatcttctgcgac and probe aggcaggtcccctagaagaagaactcc for 3.5 kb HBV RNA species quantification. Droplet formation was carried out using a QX200 AutoDG droplet generator. Subsequent amplifications were performed in the C1000 Touch™deep-well thermal cycler (Bio-Rad) with a ramp rate of 2 °C/s and the lid heated to 105 °C, according to the Bio-Rad recommendations. First, the enzyme was activated at 95 °C for 10 min followed by 40 cycles of denaturation at 94 °C for 30 s and 60 °C for 1 min. The enzyme was deactivated at 98 °C for 10 min and the reaction was kept at 4 °C.

Quantification of HBV RNA with the Roche MWF investigational assay

The Roche Manual Workflow (MWF) is a manual version developed for research purposes of the cobas® 6800/8800 Automated Investigational Assay (IA) for the detection and quantification of circulating HBV RNAs in chronic HBV patients.[17,18] MWF uses identical chemistry and PCR primers/probe sets as the cobas® 6800/8800 automated IA but does not require high throughput and automation. Results obtained with this manual assay were highly correlated with those obtained with cobas HBV RNA. HBV RNA assay characteristics are the following: i) linear results between 10 and 10^7 copies/ml in clinical samples of several HBV genotypes; ii) precision and reproducibility with standard deviation below 0.15 $\log_{10}$ copies/ml and coefficients of variation below 5% throughout the linear range; minimal impact (<0.3 $\log_{10}$ copies/ml) of HBV DNA on HBV RNA quantification at DNA:RNA ratios of up to approximately 10^6. HBV RNA quantification was calibrated using a synthetic arRNA containing 435 bp derived from the 3' end of HBV pgRNA, packaged in MS2-phage (kindly provided by Roche Diagnostics, Pleasanton, CA). arRNA was quantified by ddPCR (Bio-Rad) using a primers/probe set in the precore/core region. HBV RNA quantification with the MWF was performed according to the manufacturer's protocol.

Iodixanol/sucrose density gradient centrifugation

300 ml of supernatants were collected and centrifuged at 1,500 × g for 15 min at room temperature. Cellular debris were removed by filtration through a 0.22 µm filter (Merck Millipore, KGaA, Darmstadt, Germany). Viral particles were concentrated using Amicon® Pro Purification System with 100 kDa filter. Concentrated supernatants were then ultracentrifuged at 110,000 × g for 2h at 4 °C. The pellets were washed with 8 ml of PBS and a second ultracentrifugation was performed at 110,000 × g for 2h at 4 °C. Pellets were resuspended in 2.5 ml of 10% iodixanol solution. 10%, 20%, 30% and 40% iodixanol solutions were prepared by mixing Optiprep™ (Axis Shield) with buffer containing 0.25 M sucrose, 10 mM Tris at pH 8.0, and 1 mM EDTA, with final pH 7.4. Resuspended EVs pellets were layered on the top of the gradient and then subjected to ultracentrifugation in a SW41–Ti Rotor tube (Beckman) for 6 h at 4 °C at 110,000 × g. Twelve fractions of 1 ml were recovered and analyzed separately.

Viral protein detection

ELISA tests for HBeAg and HBsAg detection in cell supernatants were performed according to the manufacturer's protocol using the CLIA kits from Autobio Diagnostic.

Viral proteins were detected in the density gradient fractions by Western blot. Fractions were mixed with Laemmli buffer and heated at 95 °C for 5 min. Proteins were migrated in 4-20% mini-PROTEAN@ TGX stain-FreeTM Precast Gel (Bio-Rab Laboratories) and transferred onto a nitrocellulose membrane (Bio-Rab Laboratories). Membranes were blocked 1 h with 5% milk or BSA (Sigma) in TBS (1 × Tris Buffer Saline (Sigma)) and stained with primary antibodies in blocking buffer overnight at 4 °C and stained with HRP-conjugated secondary antibodies (1/50,000) for 1 h at room temperature. Detection was performed using Clarity or Clarity Max Western ECL and the ChemiDoc XRS system (Bio-Rad).

5'RACE analysis

5' rapid amplification of cDNA ends (5'RACE) was performed as previously described in.[26] Briefly, RNAs were isolated using a guanidinium thiocyanate–phenol–chloroform extraction protocol (TRI reagent (Sigma)). 5'RACE was essentially performed as described in the GeneRacer Kit manual (ThermoFisher Scientific) except Tobacco Acid Pyrophosphatase was substituted by RNA 5' Pyrophosphohydrolase (New England Biolabs) and SuperScript reverse transcriptase III by SuperScript reverse transcriptase IV (ThermoFisher Scientific). The reverse transcription reaction was performed using 3' HBV specific Gsp1 primer 5'-TTAGGCAGAGGTGAAAAAAGTTG-3'. For the 5'RACE PCR reaction Prime Star super mix DNA Polymerase (TAKARA bio), GeneRacer 5' primer and HBV specific nested primer Gsp1 5'- TTAGGCAGAGGTGAAAAAAGTTG -3' were used. 5'RACE PCR was run in a C100 Touch thermocycler (Bio-Rad) using the following PCR program: initial denaturation step 98 °C 3 min >5x (98 °C 10 s; 72 °C 3 min) >5x (98 °C 10 s; 70 °C 3 min) >25x (98 °C 10 s; 64,4 20 s; 72 °C 3min) >72 °C 10min.

MinION sequencing, data processing and bioinformatics analysis

5'RACE Samples were further prepared individually following the recommendations of the Nanopore protocol for the SQK-PBK004. Base calling was performed with Guppy (v. 6.4.6)[27] and simultaneously filtered for base called reads with PHRED quality >7 and trimmed for technical adapters. Further quality controls of reads before and after mapping were performed with pycoQC (v. 2.5.2).[28] Median PHRED score is 11.455 for passed reads and

99.6 % of reads are conserved with acceptable quality. Raw reads were trimmed for technical adapters using Porechop (https://github.com/rrwick/Porechop). Reads containing 5'RACE adapter sequence were extracted with seqkit (v. 2.1.0)[29] using the grep function, with 10% mismatch allowed due to error rate of Oxford MinION Nanopore sequencing. Reads were trimmed for the 5'RACE adapter sequence using cutadapt (v. 3.5).[30] Trimmed reads were mapped to the genomic sequence corresponding to the full-length preCore RNA sequence (from the transcription start site [TSS] to the canonical HBV poly-A, derived from HBV genome, strain ayw, NC_003977.2) with Minimap2 (v. 2.21-r1071)[31,32] using the spliced long reads option (-ax splice). Sam files were filtered, binarized and sorted with Samtools.[33] Start positions were extracted from genomic alignment using Samtools, splice junctions were identified using Nanosplicer (v. 1.0),[34] then quantification and graphical representations were produced using R scripts (v. 4.1.2).

Statistics

Statistical tests were performed using GraphPad Prism v7.05 software. Pairwise comparisons were analyzed using the Wilcoxon signed-rank test for non-parametric data and paired t test for parametric data. The comparison of more than three groups was performed using the Kruskal-Wallis test. Shapiro-Wilk normality test was performed to assess the normality of the parameter's distribution (GraphPad prism). Differences were considered significant at confidence levels greater than 95% (p ≤0.05).

Results

Generation of cell lines carrying modified HBV genomes and inverted DNA/RNA secretion

To generate a continuous source of HBV RNA to be used to calibrate HBV RNA quantification assays, we established a stable clonal cell line carrying a modified HBV genome and producing and secreting RNA-containing viral particles similar to those found in patient sera. To this aim we selected three candidate mutations in the catalytic and TP domains of viral polymerase, and in the ε-loop of the pgRNA that are expected, alone or in combination, to impact on pgRNA encapsidation, reverse transcription, relaxed circular DNA (rcDNA) formation and global viral production (Fig. 1A).

The YMDD motif is in the catalytic site of the HBV Pol and mutations in this motif are involved in resistance to anti-HBV nucleoside analogues.[35–38] Different mutations in the YMDD domain also result in a 20% to 90% decrease in viral DNA synthesis.[36,38] The specific mutations selected for this study change the YMDD motif in YMAA (D540A/D541A).

The Polymerase tyrosine 63 residue (Y63) in the TP domain is required for the priming of reverse transcription by providing the hydroxyl group necessary for the covalent binding between the first nucleotide of the negative strand of DNA and the viral polymerase.[39–41] Mutation of tyrosine 63 to a phenylalanine (Y63F) has been shown to result in a strong increase in HBV RNA virions containing pgRNA.[42] The A1G or Q18R mutation in the pgRNA stem loop structure of the encapsidation signal has been shown to induce a drastic decrease in viral DNA synthesis by interfering with the priming of reverse transcription.[43]

A WT and five mutant 1.1 unit length HBV genotype D genomes (YMAA (1); Y63F (2); YMAA + Y63F (3); A1G + YMAA (4); A1G + Y63F (5)) were synthesized and cloned into a pTriEX vector plasmid (Fig. 1B). After transfection and blasticidin selection in Huh7 cells, the six polyclonal cell lines were analyzed for HBV DNA and HBV RNA in cell extracts and cell supernatants. The YMAA + Y63F (3) and A1G + Y63F (5) polyclonal cell lines displaying the desired secretory phenotype with an inversion of the HBV DNA/RNA ratio in cell supernatants, lower DNA secretion and lower intra-experiment variability (*e.g*. SD values) were selected for the generation of clonal cell lines (Fig. 1C).

Secretory phenotype of selected clonal cells lines

Next, the WT, (3) YMAA + Y63F and (5) A1G + Y63F polyclonal cell lines were plated at low density for colony formation. After 20 days of blasticidin selection, 24 clones for each polyclonal cell line were isolated, expanded and phenotyped for HBV DNA/RNA secretion. Clones Huh7-WT18 (carrying the integrated WT HBV genome of genotype D), Huh7-5D21 (carrying a mutation in both the ε-loop of the pgRNA (A1G) and the TP domain of the polymerase (Y63F), see Fig. 1A), Huh7-3D29 and Huh7-3D35 (carrying the mutation in the TP domain of the polymerase (Y63F) and the catalytic site of the HBV polymerase (YMAA), see Fig. 1A), were selected based on cell growth, HBV RNA/DNA ratio and HBV RNA production (data not shown) and carried on for further characterization and expansion. At 9 days post plating, Huh7-WT18 cells secreted 15-fold more HBV DNA than 3.5 kb HBV RNA (1.08×10^7 copies of HBV DNA/ml *vs*. 0.7 $\times 10^6$ copies of 3.5 kb HBV RNA/ml). Huh7-5D21, Huh7-3D29 and Huh7-3D35 clonal cell lines showed high HBV RNA levels compared to HBV DNA levels in cell supernatant, with an inversion of the secreted HBV DNA/RNA ratio (Fig. 2, left panel). 3.5 kb HBV RNA secretion levels were 1.0×10^7 copies/ml in Huh7-3D35 cells, 1.6×10^7 copies/ml in Huh7-5D21 cells and 6.02×10^7 copies/ml in Huh7-3D29 cells. All three mutant clonal cell lines secreted more 3.5 kb HBV RNA than the Huh7-WT18 WT clone: 90x higher in Huh7-3D29, 27x higher in Huh7-5D21 cells and 16x higher in Huh7-3D35 cells. Notably, lamivudine treatment for 9 days resulted in a sharp reduction of secreted HBV DNA in Huh7-WT18 WT cells, a further reduction, but not a complete abrogation, of secreted HBV DNA in Huh7-3D29 cells (Fig. S1, black columns) and a slight increase in the levels of HBV DNA secreted in both the HBV RNA WT the 3D29 cells (Fig. S1, grey columns).

After a first freeze/thaw cycle (3 passages), the Huh7-5D21 and Huh7-3D29 mutant cell lines kept their RNA secretory phenotype (Fig. 2, middle panel). A decrease in secretion of all HBV nucleic acids was observed over the passages without a significant change in the HBV RNA/DNA ratio. Huh7-3D29 cells showed a 34% reduction of 3.5 kb HBV RNA secretion but kept the higher RNA/DNA ratio. After a second freeze/thaw cycle (3-4 passages), the HBV RNA secretory phenotype was conserved (Fig. 2, right panel). The Huh7-3D29 cell line again showed a higher RNA/DNA ratio with the lower residual HBV DNA (2.44×10^7 RNA copies/ml *vs*. 2.87×10^5 DNA copies/ml). Finally, the secretory phenotype of the Huh7-3D29 clone is consistent between vials issued from the same culture passage (Fig. S2).

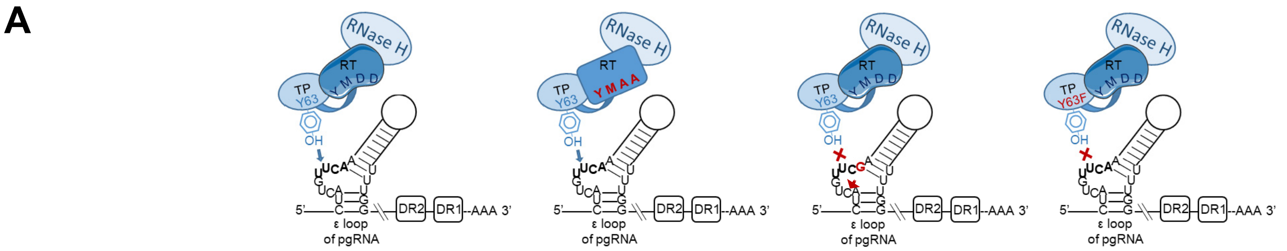

	WT	YMAA(1)	A1G(2)	Y63F(3)
DNA accumulation	100%	↘(4)	<18%	<10%
pgRNA encapsidation	100%	≈100%	≈100%	≈100%
In vitro priming	100%	↘(4)	↘(4)	20%

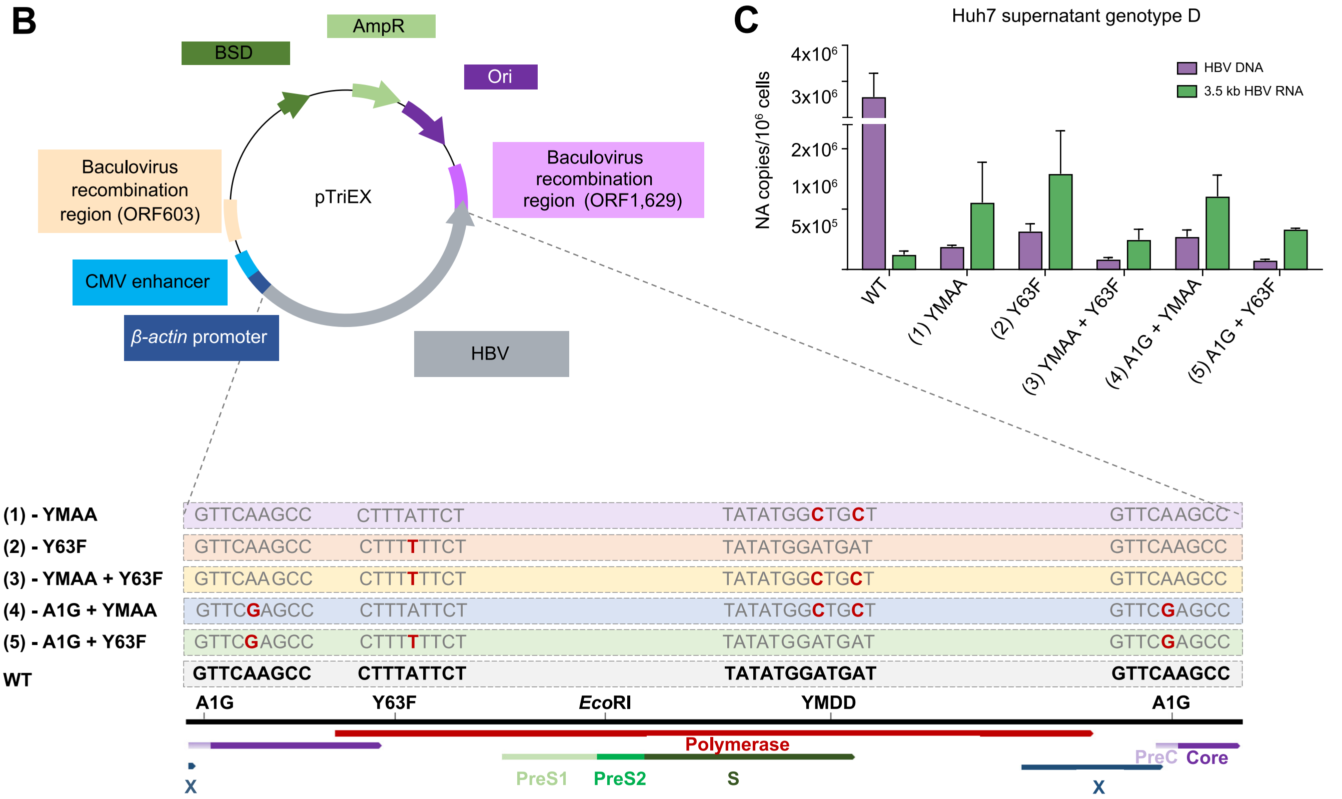

Fig. 1. Strategies to generate an HBV RNA standard. (A) Viral mutations selected for the generation of cell lines and their expected effect on viral parameters. 1 [35–38]; 2 [43]; 3 [39–41] 4 ↘: decrease, (B) pTriEX plasmid based plasmids containing one of the five 1.1-unit length HBV genotype D genomes (YMAA (1); Y63F (2); YMAA + Y63F (3); A1G + YMAA (4); A1G + Y63F (5) or the 1.1 WT HBV genome. (C) Two-step ddPCR quantification of HBV DNA and 3.5 kb HBV RNA species in the supernatants of the HBV-Huh7 polyclonal cell lines. Error bars represent standard deviation of 3 technical replicates from each of 3 distinct cell passages, which served as biological replicates. ddPCR, digital droplet PCR; NA, nucleic acid; WT, wild-type.

Characterization of the HBV RNAs secreted by the Huh7-3D29 clonal cell line

Although the majority of circulating HBV RNAs are represented by the pgRNA-containing viral particle,[26,42,44–48] increasing evidence suggests the presence of additional RNA species and variable amounts of circulating HBV RNAs might be carried in small extracellular vesicles (sEVs).[49–51] To characterize the HBV RNA species present in Huh7-WT18, Huh7-3D29, and Huh7-3D35 cell supernatants and their compartmentalization, we performed iodixanol/sucrose density gradient ultracentrifugation[52] followed by HBsAg quantification by ELISA, HBV DNA and HBV RNA quantification by ddPCR, and HBc and CD9 sEV marker[53] detection by western blot (Fig. 3). In Huh7-WT18 cell supernatants (Fig. 3A), HBsAg was detected in fractions 4-8 of

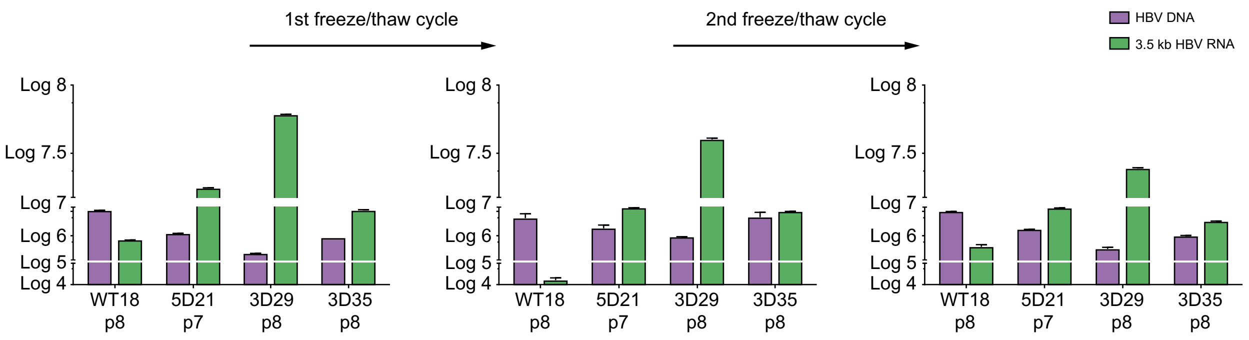

Fig. 2. HBV nucleic acid secretion profiles of selected HBV-Huh7 stable clones. Two-step ddPCR quantification of secreted HBV DNA and 3.5 kb HBV RNA species from Huh7 clonal cell lines at different passages and freezing/thawing cycles. WT = WT genotype D HBV. 3D = genotype D HBV genome carrying the ε-loop A1G mutation and the HBV polymerase catalytic site YMAA mutation. 5D = genotype D HBV genome carrying the ε-loop A1G mutation and the Y63F mutation in the TP domain of the HBV polymerase. The values following the WT, 3D and 5D label indicate the clone number. The *p* followed by a value correspond to the number of passages in cell culture. Error bars represent standard deviation of two technical replicates from each of three distinct cell passages, which served as biological replicates. ddPCR, digital droplet PCR; WT, wild-type.

the density gradient, with one major peak in fraction 7 (Fig. 3A, upper panel), and HBc was detected from fractions 7 to 9 (Fig. 3A, lower panels). HBV DNA was found in almost all the fractions except in fractions 1 and 2, with a peak in fraction 8 (Fig. 3A, upper panel). HBV RNA was found in fractions 6 to 9 (Fig. 3A, upper panel). Fractions 3 and 4 contain sEVs, identified by the detection of the CD9 membrane protein[53] (Fig. 3A, lower panels). Notably, in Huh7-WT18 cell supernatants the sEVs contained only HBV DNA (Fig. 3A, upper panel). The detection of both HBsAg and HBc identifies viral particles, while the detection of HBc only identifies naked capsids. Fractions 7 and 8, positive for both HBsAg and HBc, were positive for both HBV DNA (virions) and HBV RNA (RNA particles) (Fig. 3, upper panel). Fraction 9, positive for HBc only, contained only low levels of HBV RNA compared to HBV DNA, indicating that naked capsids mostly carry HBV DNA in WT cells (Fig. 3A, upper panel). In Huh7-3D29 cell supernatants, HBV RNA was found in RNA particles (fraction 7 to 8) and in naked capsids (fraction 9) while the residual HBV DNA peak was found in virions (fraction 7) (Fig. 3B, upper panel). Compared to the Huh7-WT18 cell, a larger proportion of HBV RNA was found in naked capsids. This observation is consistent with the reduction of HBV DNA synthesis in the immature capsid imposed by the HBV mutations and the resulting blockade of capsid maturation. Regarding the distribution of HBsAg, HBc and CD9 in the different fractions, no differences were noticed compared to the WT clone.

To refine the characterization of the RNA species secreted by Huh7-3D29 cells, we have performed 5'RACE coupled with single molecule long-read Nanopore sequencing. Gel electrophoresis analysis of the 5'RACE products from Huh7-3D29 cells and supernatants shows that only 3.5 kb RNA species were detected in

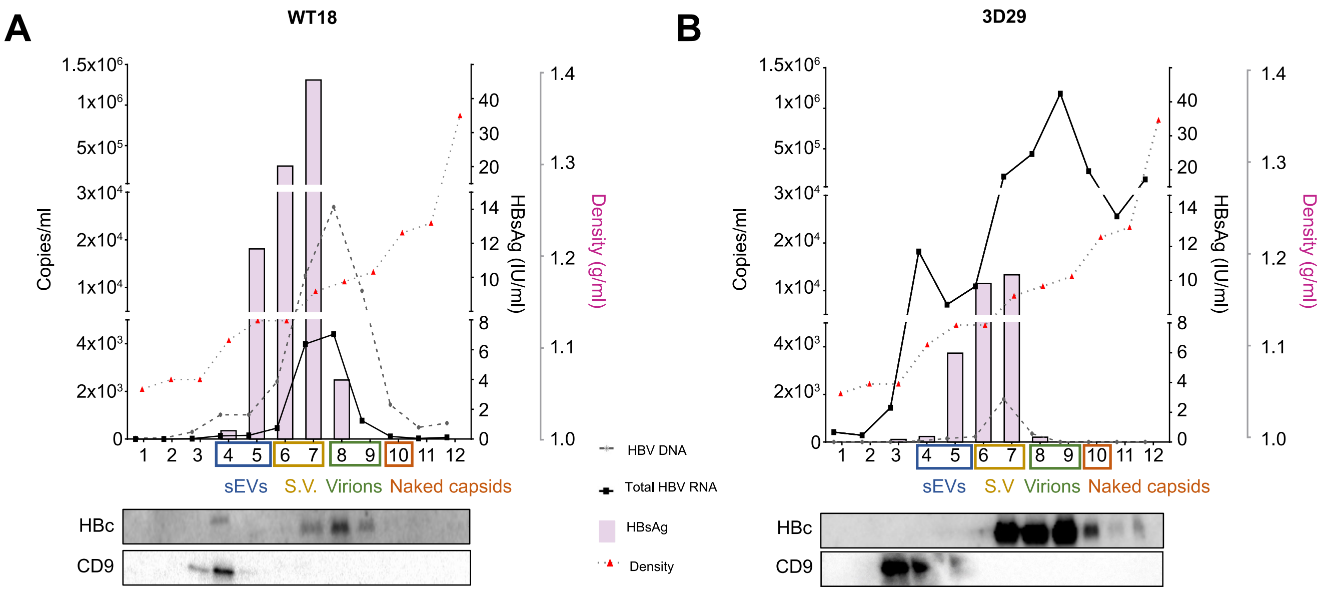

Fig. 3. Sucrose/iodixanol gradient characterization of HBV nucleic acids secreted in HBV-Huh7 3D29 clone culture supernatant. *Upper panels*: Distribution of hepatitis B viral particle-associated antigens and DNA/RNA in sucrose/iodixanol gradient for WT18 (A) and 3D29 (B) clonal cell lines. Total HBV DNA and total HBV RNA were quantified in the sucrose/iodixanol gradient fraction by two-step ddPCR, as detailed in the Methods section. *Lower panels*: Western Blot detection of viral HBc protein and CD9 sEV marker. ddPCR, digital droplet PCR; sEV, small extracellular vesicle; WT, wild-type.

cell supernatants (Fig. 4A). Nanopore sequencing of the 5'RACE products shows that: a) the secreted HBV RNA is identical to the HBV genome used for the establishment of the different cell lines; and b) the majority of HBV RNAs detected start at the 5'end of pgRNA and pgRNA-derived spliced RNAs; b) lower amounts of several HBx RNA species are present; c) PreS/S RNAs and PreS/S-derived spliced RNA are barely detectable (Fig. 4B, upper panel). The proportion of spliced transcripts from the 3.5 kb RNA TSS is low (0.84%), with a relative enrichment of SP1 (84.2%) as the prominent species (Fig. S3A). The profile of HBV RNAs detected in the cellular fraction was very different with all canonical HBV RNA transcripts present and a majority of PreS1/PreS2/S transcripts. Spliced RNAs represent 1.77% of all transcripts derived from the 3.5 kb RNA TSS (Fig. S3B). SP1 accounts for

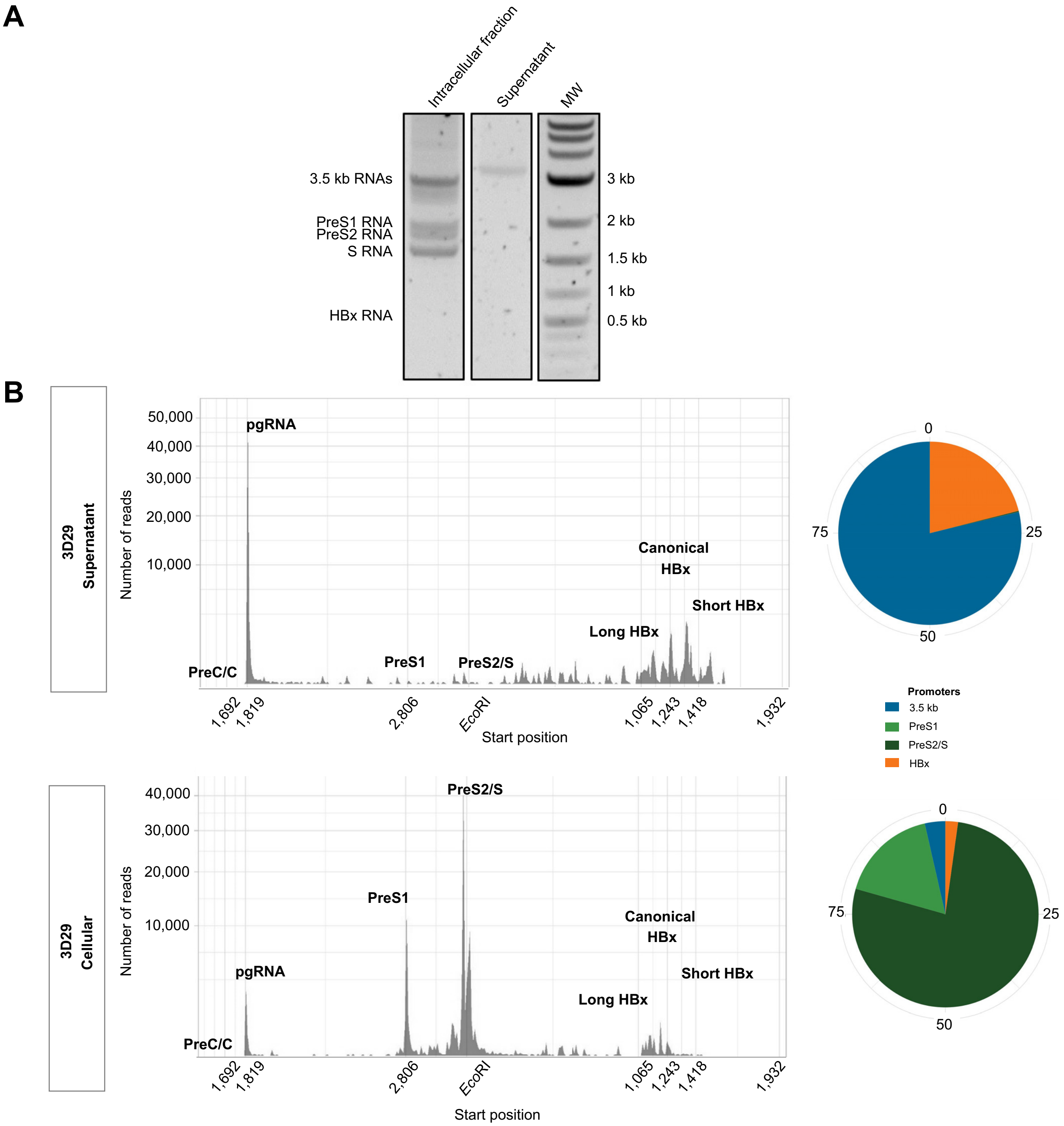

Fig. 4. Characterization of HBV RNAs in Huh7-3D29 cell culture supernatant. (A) PAGE analysis of the 5'RACE performed on cell extracts and culture supernatants of the Huh7-3D29 clone. Canonical HBV RNA positions are indicated on the left. (B) Nanopore sequencing of the 5'RACE products in the cell culture supernatants (*upper panel*) and cellular extract of Huh7-3D29 cells (*lower panel*). 85,497 and 70,588 reads were generated for the Huh7-3D29 cellular extract and cell culture supernatants, respectively. *Left panels* are the number of reads according to their starting position after alignment on the HBV genome. *Right panels* indicate the proportion (%) of reads associated with each HBV canonical promoter. MW, molecular weight; 5'RACE, 5' rapid amplification of cDNA ends.

27.8% of all the spliced HBV RNAs starting at the 3.5 kb RNA TSS and 0.7% of all 3.5 kb RNAs, with several other SP variants represented (Fig. S3B). Altogether, the coupled 5'RACE – Nanopore sequencing results support the notion that the Huh7-3D29 HBV RNA-containing supernatants could be used to standardize RUO HBV RNA investigational assays[16–18] and in house PCR-based HBV RNA assays[13] aimed at quantifying HBV pgRNA.

Use of RNA-containing supernatant from Huh7-3D29 cells to standardize the manual version (MWF) of the HBV RNA Roche IA

Ten-fold dilution (from 10^5 to 10^1 copies/ml) of HBV arRNA (Roche Diagnostics, Pleasanton, CA)[17,18] and Huh7-3D29 supernatants, processed as described in the Materials and Methods, have been quantified using the HBV RNA Roche MWF kit.[17,18] The amplification curves obtained after quantitative reverse-transcription PCR from the MWF showed very similar profiles (Fig. 5A, left and right panels). Furthermore, no significant difference was found between CTs of the 10-fold diluted arRNA and Huh7-3D29 cell supernatants (Fig. 5A, middle panel). Next, we compared the performance of serial dilutions of cell supernatants from three Huh7-3D29 passages and of the HBV arRNA using an *in house* ddPCR (see Materials and Methods) (Figs 5B and S6). HBV RNA quantification of Huh7-3D29 cell supernatants with and without a DNase treatment showed no significant difference (Fig. 5C). The HBV RNA Roche MWF kit used here is the manual version of the Roche cobas® 6800/8800 automated IA for HBV RNA quantification that does not include a DNase treatment in its workflow.[17,18] The lack of difference in RNA quantification with or without the DNase treatments validates the use of Huh7-3D29 supernatants as an RNA standard for both the HBV RNA MWF and the cobas® 6800/8800 automated assay.

Discussion

Circulating HBV RNAs have emerged as a new biomarker that reflects the transcriptional activity of cccDNA[12,13] and may help monitor both CHB infection[11,14,15] and the antiviral effect of new anti-HBV therapeutics. Several *in house* PCR-based assays for circulating HBV RNAs have been described[13] and two investigational RUO assays have been developed by industry.[16–18] These assays use different DNA (*e.g.*, the WHO HBV DNA standard[19–24]) or RNA (*e.g.*, synthetic arRNAs[54]) to calibrate circulating HBV RNA quantification.[17,18] The use of HBV DNA as a standard has been challenged[25] and arRNA synthesis is unpractical and costly for routine use. We describe here the development and characterization of an HBV RNA standard suitable for all PCR-based HBV RNA quantification assays. From a theoretical point of view, an HBV RNA standard could be produced by: i) extracting HBV RNA from patients' sera/plasma; ii) *in vitro* transcription of pregenomic or subgenomic HBV RNAs; in both cases the RNA would be naked (*e.g.* not in viral particles, capsids or extracellular vesicles/exosomes) and neither approach would allow for standardization of the extraction step; if sub-genomic RNAs are used, the standard may not be applicable to all HBV RNA detection strategies, especially if PCR target sequences are located in the 5' region of the 3.5 kb RNAs; iii) engineering HBV cell lines to exclusively, or predominantly, produce RNA-containing viral particles similar to those found in patients' sera. These different approaches must balance quantitative yield, convenient production setting and costs with the need to avoid, or at least minimize, the impact of residual HBV DNA on the HBV RNA assay. Here, we have chosen to generate a stable cell line carrying an integrated HBV genome with mutations that would allow pgRNA encapsidation but prevent its reverse transcription into rcDNA due to the expression of a non-functional viral polymerase. Three candidate mutations (YMAA, Y63F and A1G) have been selected according to their expected impact on pgRNA encapsidation, reverse transcription, rcDNA formation, global viral production[35–43] and their capability, alone or in combination, to result in an inversion of the HBV DNA/RNA ratio with a higher proportion of pgRNA than rcDNA in cell supernatants.

We have down-selected the best Huh7-derived clonal cell line carrying the double mutation in the catalytic site of the polymerase (YMAA mutation) and the TP domain (Y63F) of the polymerase (Huh7-3D29) that displays an inversion of the secreted HBV DNA/RNA ratio and maintains the desired RNA secretory phenotype over several freeze-thaw and amplification cycles with minimal residual HBV DNA secretion. If one considers the performing characteristics of either Roche MWF assay used in this study or the related HBV RNA cobas® 6800/8800 automated investigational assay,[17,18] both tolerating ~10^6 DNA:RNA ratios without losing RNA specificity and linearity, the HBV RNA standard produced and secreted by the Huh7-3D29 cell line fulfill this criterion. Notably, HBV RNA quantification of Huh7-3D29 supernatants with the Roche MWF showed no significant difference with and without DNase treatment, supporting the use of the Huh7-3D29-derived HBV RNA standard with both automated and manual assays. Additional observations validate the use of Huh7-3D29 cell supernatants as an HBV RNA standard. First, the quantification of HBV RNAs with the Roche MWF of standard curves obtained from 10-fold dilution of Huh7-3D29 supernatants and a synthetic HBV arRNA showed the same profile. Second, the CTs obtained with both standards showed no significant differences. More importantly, the results of iodixanol/sucrose gradients and 5'RACE coupled with single molecule long-read Nanopore sequencing indicate that, as observed in patients with CHB, the majority of secreted HBV RNAs are full length or spliced 3.5 kb species in naked capsid or virion-like particles. These results support the relevance of the Huh7-3D29 supernatants as a standard to quantify HBV RNAs with investigational RUO assays and *in house* PCR-based HBV RNA assays.

The requirements for a WHO IS is a lyophilized preparation with proven long-term stability that is available in adequate quantities to last several years.[22] All viral nucleic acid WHO ISs have been obtained from serum preparation. Therefore, the production of an international HBV RNA standard from a stable clonal cell line would be a novelty and would guarantee sustained access. The Huh7-3D29 cell line is deposited in the CNCM repository (https://www.pasteur.fr/fr/sante-publique/centre-ressources-biologiques/collection-nationale-culturesmicro-organismes-cncm) and constitutes a potentially unlimited source of HBV RNA standard. Twenty very early passage vials of Huh7-3D29 are conserved in liquid nitrogen at the INSERM Lyon CAT Biobank DC-2008-235 (Ethical Commitee CPP 11/040) to guarantee a steady supply of the HBV RNA standard to support its validation and diffusion. One 175 cm^2 flask of Huh7-3D29 cell provides, in 9

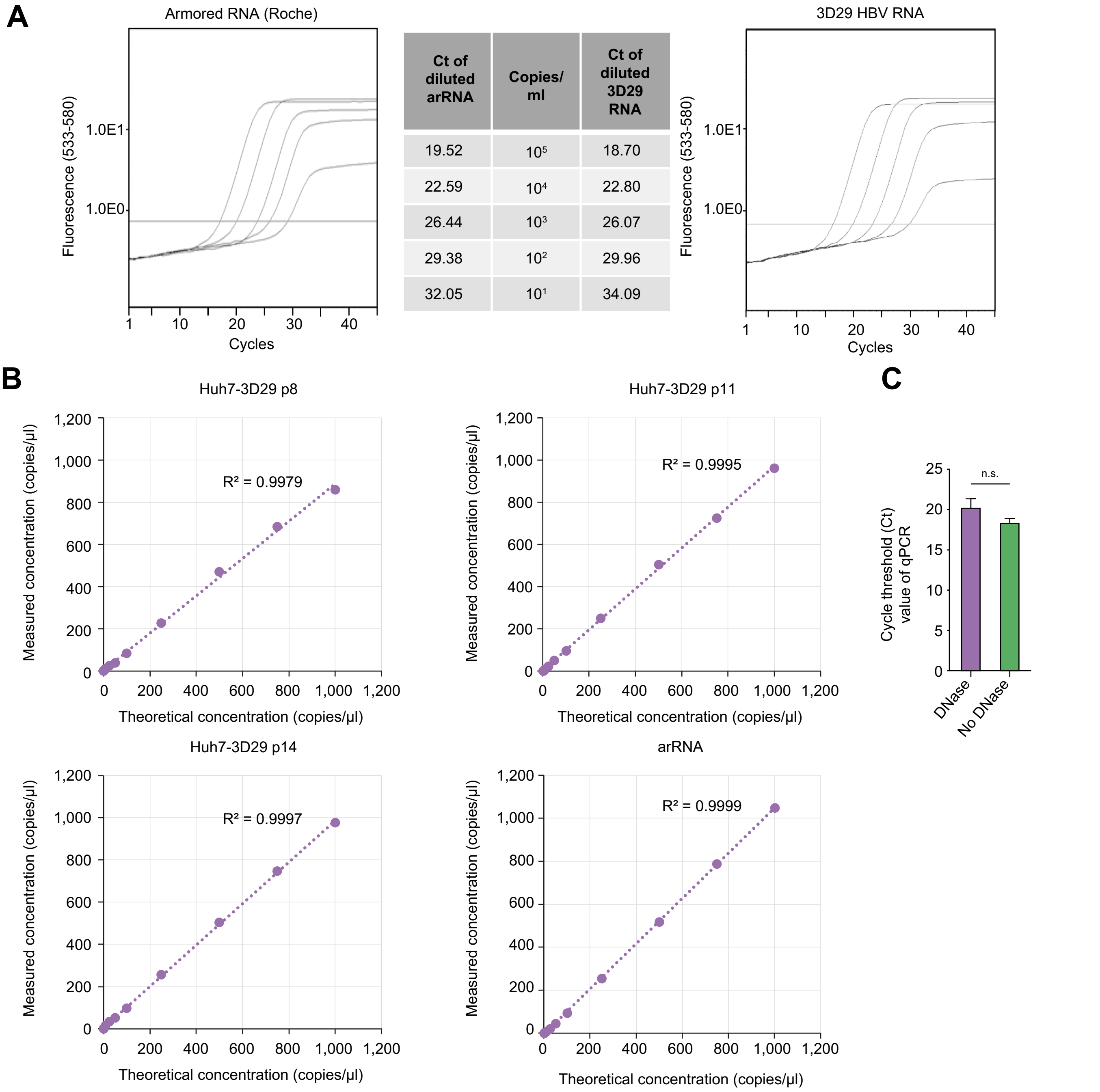

Fig. 5. Validation of Huh7-3D29 cell culture supernatant as a standard for HBV RNA quantification assays. (A) The comparison of 10-fold dilutions of Huh7-3D29 supernatant and armored RNA performance did not show significant differences (paired t test, p = 0.54). Amplification curves of the arRNA (*left panel*) and 3D29 HBV RNA (*right panel*) in the Roche Manual workflow assay. *Middle panel*: Ct values and corresponding concentration in copies/ml. Validation of samples parametric distribution by Shapiro-Wilk test. (B) Performance of serial dilutions of cell supernatants from three Huh7-3D29 passages and of the HBV arRNA in an *in house* ddPCR. (C) Huh7-3D29 HBV RNA quantification with and without DNAse treatment (Wilcoxon test, p = 0.25). Validation of samples non-parametric distribution by Shapiro-Wilk test. Error bars represent standard deviation of two technical replicates from each of three distinct cell passages, which served as biological replicates. arRNA, armored RNA; ddPCR, digital droplet PCR.

days, enough material for 1,300 standard curves and the production can easily be scaled up. As a comparison, the HBV nucleic acid WHO IS produced 2,000 aliquots of 0.5 ml with a concentration of 1 × 10^6 IU/ml and the second, third and fourth HBV WHO standard productions were in the same range.[19,20,22] The next steps for developing the Huh7-3D29 HBV RNA standard into an internationally established standard or a WHO-endorsed standard will include i) the lyophilization of the standard material, ii) the evaluation of its performance by a large panel of laboratories, iii) the analysis of its performance by the independent expert committee on biological standardization of the WHO.[21]

Altogether, the establishment of the Huh7-3D29 cell line with a continuous production of HBV RNA-containing supernatants will allow us to share this viral RNA material with research and diagnostic laboratories to standardize HBV RNA quantification and assist the evaluation of emerging HBV cure strategies.

Affiliations

[1]IHU Lyon, Lyon Hepatology Institute, Lyon, France; [2]Cancer Research Center of Lyon (CRCL), UMR Inserm U1052 / CNRS 5286, Lyon, France; [3]University of Lyon, University Claude Bernard Lyon 1, 69008 Lyon, France; [4]Department of Hepatology, Hospices Civils de Lyon, France; [5]Laboratoire de Virologie, Institut des Agents Infectieux, Hospices Civils de Lyon, Lyon, France; [6]Department of Internal Medicine, SCIAC and the IIT Center for Life Nanoscience, Sapienza University, Rome, Italy

Abbreviations

arRNA, armored RNA; cccDNA, covalently closed circular DNA; CHB, chronic hepatitis B; ddPCR, droplet digital PCR; HBsAg, hepatitis B surface antigen; IA, investigational assays; IFN, interferon; IS, International Standard; MWF, Manual Workflow; NMEs, new molecular entities; NUCs, nucleos(t)side analogues; pgRNA, pregenomic RNA; 5'RACE, 5' rapid amplification of cDNA ends; RUO, research use only; sEVs, small extracellular vesicles; WHO, World Health Organization; WT, wild-type.

Financial support

This work was supported by a public grant overseen by the French National Research Agency (ANR) as part of the second "Investissements d'Avenir" program (reference: ANR-17-RHUS-0003).

Conflict of interest

The authors disclose no conflicts.

Please refer to the accompanying ICMJE disclosure forms for further details.

Authors' contributions

Conceptualization: ML and FZ; Funding acquisition: ML and FZ; Formal Analysis: AP, XG, MLP, BT; Investigation: AP, FCT, DB, MLP, HT, FB, CS, BT; Methodology: AP, DB, XG; Resources: MLP, ML, FZ; Supervision: ML, BT, FZ; Visualization: AP, ML; Writing – original draft: AP, ML; Writing – review & editing: all authors.

Data availability statement

Data are available at reasonable request to the corresponding author.

Acknowledgements

Graphical abstract created with BioRender.com.

Supplementary data

Supplementary data to this article can be found online at https://doi.org/10.1016/j.jhepr.2024.101124.

References

Author names in bold designate shared co-first authorship

[1] Global hepatitis report, 2017. https://www.who.int/publications-detail-redirect/global-hepatitis-report-2017. [Accessed 21 February 2022].
[2] Lampertico P, Agarwal K, Berg T, et al. EASL 2017 Clinical Practice Guidelines on the management of hepatitis B virus infection. J Hepatol 2017;67:370–398. https://doi.org/10.1016/j.jhep.2017.03.021.
[3] Lim SG, Baumert TF, Boni C, et al. The scientific basis of combination therapy for chronic hepatitis B functional cure. Nat Rev Gastroenterol Hepatol 2023. https://doi.org/10.1038/s41575-022-00724-5.
[4] Ghany MG, Buti M, Lampertico P, et al. 2022 AASLD-EASL HBV-HDV Treatment Endpoints Conference Faculty. Guidance on treatment endpoints and study design for clinical trials aiming to achieve cure in chronic hepatitis B and D: report from the 2022 AASLD-EASL HBV/HDV treatment endpoints conference. J Hepatol 2023. https://doi.org/10.1016/j.jhep.2023.06.002. S0168-8278(23)00417-8.
[5] Fanning GC, Zoulim F, Hou J, et al. Therapeutic strategies for hepatitis B virus infection: towards a cure. Nat Rev Drug Discov 2019;18:827–844. https://doi.org/10.1038/s41573-019-0037-0.
[6] Nassal M. HBV cccDNA: viral persistence reservoir and key obstacle for a cure of chronic hepatitis B. Gut 2015;64:1972–1984. https://doi.org/10.1136/gutjnl-2015-309809.
[7] Charre C, Levrero M, Zoulim F, et al. Non-invasive biomarkers for chronic hepatitis B virus infection management. Antivir Res 2019;169:104553. https://doi.org/10.1016/j.antiviral.2019.104553.
[8] Allweiss L, Testoni B, Yu M, et al. Quantification of the hepatitis B virus cccDNA: evidence-based guidelines for monitoring the key obstacle of HBV cure. Gut 2023;72:972–983. https://doi.org/10.1136/gutjnl-2022-328380.
[9] **Wooddell CI**, **Yuen M-F**, Chan HL-Y, et al. RNAi-based treatment of chronically infected patients and chimpanzees reveals that integrated hepatitis B virus DNA is a source of HBsAg. Sci Transl Med 2017;9. https://doi.org/10.1126/scitranslmed.aan0241.
[10] Kramvis A, Chang K-M, Dandri M, et al. A roadmap for serum biomarkers for hepatitis B virus: current status and future outlook. Nat Rev Gastroenterol Hepatol 2022;19:727–745. https://doi.org/10.1038/s41575-022-00649-z.
[11] **Fan R**, **Peng J**, **Xie Q**, et al. Combining HBV RNA and hepatitis B core-related antigen: guidance for safely stopping nucleos(t)ide analogues in HBeAg-positive chronic hepatitis B patients. J Infect Dis 2020. https://doi.org/10.1093/infdis/jiaa136.
[12] Giersch K, Allweiss L, Volz T, et al. Serum HBV pgRNA as a clinical marker for cccDNA activity. J Hepatol 2017;66:460–462. https://doi.org/10.1016/j.jhep.2016.09.028.
[13] Liu S, Zhou B, Valdes JD, et al. Serum hepatitis B virus RNA: a new potential biomarker for chronic hepatitis B virus infection. Hepatol Baltim Md 2019;69:1816–1827. https://doi.org/10.1002/hep.30325.
[14] Carey I, Gersch J, Wang B, et al. Pre-genomic HBV RNA and HBcrAg predict outcomes in HBeAg negative chronic hepatitis B patients suppressed on nucleos(t)ide analogue therapy. Hepatol Baltim Md 2019. https://doi.org/10.1002/hep.31026.
[15] Ma G, Lou B, Lv F, et al. HBcrAgandpgRNAand the therapeutic effect inHBeAg-positive patients receiving anti-viral therapy, baseline serum HBV-RNAis a powerful predictor of response. J Viral Hepat 2020. https://doi.org/10.1111/jvh.13299.
[16] Butler EK, Gersch J, McNamara A, et al. Hepatitis B virus serum DNA andRNA levels in nucleos(t)ide analog-treated or untreated patients during chronic and acute infection. Hepatol Baltim Md 2018;68:2106–2117. https://doi.org/10.1002/hep.30082.
[17] Scholtès C, Hamilton AT, Plissonnier M-L, et al. Performance of the cobas® HBV RNA automated investigational assay for the detection and quantification of circulating HBV RNA in chronic HBV patients. J Clin Virol 2022;150–151:105150. https://doi.org/10.1016/j.jcv.2022.105150.
[18] Cortese MF, Riveiro-Barciela M, Tabernero D, et al. Standardized hepatitis B virus RNA quantification in untreated and treated chronic patients: a promising marker of infection follow-up. Microbiol Spectr 2022;10:e02149-21. https://doi.org/10.1128/spectrum.02149-21.
[19] Baylis SA, Heath AB, Chudy M, et al. An international collaborative study to establish the 2nd World Health Organization International Standard for hepatitis B virus DNA nucleic acid amplification technology-based assays. Vox Sang 2008;94:358–362. https://doi.org/10.1111/j.1423-0410.2008.01023.x.
[20] Fryer JF, Heath AB, Wilkinson DE, et al. A collaborative study to establish the 3rd WHO International Standard for hepatitis B virus for nucleic acid amplification techniques. Biol J Int Assoc Biol Stand 2017;46:57–63. https://doi.org/10.1016/j.biologicals.2016.12.003.
[21] Organization WH, Standardization Wec on B. Collaborative study to evaluate the proposed WHO 4th international standard for hepatitis B virus (HBV) DNA for nucleic acid amplification technique (NAT) based assays. 2016.
[22] Saldanha J, Gerlich W, Lelie N, et al. An international collaborative study to establish a World Health Organization international standard for hepatitis B virus DNA nucleic acid amplification techniques. Vox Sang 2001;80:63–71. https://doi.org/10.1046/j.1423-0410.2001.00003.x.
[23] Heermann K-H, Gerlich WH, Chudy M, et al. Quantitative detection of hepatitis B virus DNA in two international reference plasma preparations. J Clin Microbiol 1999;37:68–73.

[24] Jenkins A, Minhas R, Morris C, et al. Complete Genome Sequence of the WHO International Standard for Hepatitis B Virus DNA. Genome Announc 2017;5. https://doi.org/10.1128/genomeA.01576-16.
[25] Liu S, Deng R, Zhou B, et al. Is World Health Organization HBV DNA Standard Appropriate for Standardizing Serum HBV RNA Assay? Clin Infect Dis Off Publ Infect Dis Soc Am 2020. https://doi.org/10.1093/cid/ciaa1503.
[26] Stadelmayer B, Diederichs A, Chapus F, et al. Full-length 5'RACE identifies all major HBV transcripts in HBV-infected hepatocytes and patient serum. J Hepatol 2020;73:40–51. https://doi.org/10.1016/j.jhep.2020.01.028.
[27] Wick RR, Judd LM, Holt KE. Performance of neural network basecalling tools for Oxford Nanopore sequencing. Genome Biol 2019;20:129. https://doi.org/10.1186/s13059-019-1727-y.
[28] Leger A, Leonardi T. pycoQC, interactive quality control for Oxford Nanopore Sequencing. J Open Source Softw 2019;4:1236. https://doi.org/10.21105/joss.01236.
[29] Shen W, Le S, Li Y, et al. SeqKit: A Cross-Platform and Ultrafast Toolkit for FASTA/Q File Manipulation. PLOS ONE 2016;11:e0163962. https://doi.org/10.1371/journal.pone.0163962.
[30] Martin M. Cutadapt removes adapter sequences from high-throughput sequencing reads. EMBnetJournal 2011;17:10–12. https://doi.org/10.14806/ej.17.1.200.
[31] Li H. New strategies to improve minimap2 alignment accuracy. Bioinforma Oxf Engl 2021;37:4572–4574. https://doi.org/10.1093/bioinformatics/btab705.
[32] Li H. Minimap2: pairwise alignment for nucleotide sequences. Bioinformatics 2018;34:3094–3100. https://doi.org/10.1093/bioinformatics/bty191.
[33] Danecek P, Bonfield JK, Liddle J, et al. Twelve years of SAMtools and BCFtools. GigaScience 2021;10:giab008. https://doi.org/10.1093/gigascience/giab008.
[34] You Y, Clark MB, Shim H. NanoSplicer: accurate identification of splice junctions using Oxford Nanopore sequencing [n.d].
[35] Hatakeyama T, Noguchi C, Hiraga N, et al. Serum HBV RNA is a predictor of early emergence of the YMDD mutant in patients treated with lamivudine. Hepatol Baltim Md 2007;45:1179–1186. https://doi.org/10.1002/hep.21581.
[36] Lai C-L, Dienstag J, Schiff E, et al. Prevalence and clinical correlates of YMDD variants during lamivudine therapy for patients with chronic hepatitis B. Clin Infect Dis Off Publ Infect Dis Soc Am 2003;36:687–696. https://doi.org/10.1086/368083.
[37] Ono-Nita SK, Kato N, Shiratori Y, et al. YMDD motif in hepatitis B virus DNA polymerase influences on replication and lamivudine resistance: a study by in vitro full-length viral DNA transfection. Hepatol Baltim Md 1999;29:939–945. https://doi.org/10.1002/hep.510290340.
[38] Zhu Y, Curtis M, Borroto-Esoda K. The YMDD and rtA194T mutations result in decreased replication capacity in wild-type HBV as well as in HBV with precore and basal core promoter mutations. Antivir Chem Chemother 2011;22:13–22. https://doi.org/10.3851/IMP1791.
[39] Clark DN, Flanagan JM, Hu J. Mapping of functional subdomains in the terminal protein domain of hepatitis B virus polymerase. J Virol 2017;91. https://doi.org/10.1128/JVI.01785-16.
[40] Lanford RE, Notvall L, Lee H, et al. Transcomplementation of nucleotide priming and reverse transcription between independently expressed TP and RT domains of the hepatitis B virus reverse transcriptase. J Virol 1997;71:2996–3004.
[41] Zoulim F, Seeger C. Reverse transcription in hepatitis B viruses is primed by a tyrosine residue of the polymerase. J Virol 1994;68:6–13.
[42] **Wang J**, **Shen T**, **Huang X**, et al. Serum hepatitis B virus RNA is encapsidated pregenome RNA that may be associated with persistence of viral infection and rebound. J Hepatol 2016;65:700–710. https://doi.org/10.1016/j.jhep.2016.05.029.
[43] Feng H, Chen P, Zhao F, et al. Evidence for multiple distinct interactions between hepatitis B virus P protein and its cognate RNA encapsidation signal during initiation of reverse transcription. PLOS ONE 2013;8:e72798. https://doi.org/10.1371/journal.pone.0072798.
[44] Bai L, Zhang X, Kozlowski M, et al. Extracellular hepatitis B virus RNAs are heterogeneous in length and circulate as capsid-antibody complexes in addition to virions in chronic hepatitis B patients. J Virol 2018;92. https://doi.org/10.1128/JVI.00798-18.
[45] Jansen L, Kootstra NA, van Dort KA, et al. Hepatitis B virus pregenomic RNA is present in virions in plasma and is associated with a response to pegylated interferon alfa-2a and nucleos(t)ide analogues. J Infect Dis 2016;213:224–232. https://doi.org/10.1093/infdis/jiv397.
[46] Prakash K, Rydell GE, Larsson SB, et al. High serum levels of pregenomic RNA reflect frequently failing reverse transcription in hepatitis B virus particles. Virol J 2018;15:86. https://doi.org/10.1186/s12985-018-0994-7.
[47] Lam AM, Ren S, Espiritu C, et al. Hepatitis B virus capsid assembly modulators, but not nucleoside analogs, inhibit the production of extracellular pregenomic RNA and spliced RNA variants. Antimicrob Agents Chemother 2017;61. https://doi.org/10.1128/AAC.00680-17.
[48] Anderson M, Gersch J, Luk K-C, et al. Circulating pregenomic hepatitis B virus RNA is primarily full-length in chronic hepatitis B patients undergoing nucleos(t)ide analogue therapy. Clin Infect Dis Off Publ Infect Dis Soc Am 2021;72:2029–2031. https://doi.org/10.1093/cid/ciaa1015.
[49] **Kouwaki T**, **Fukushima Y**, Daito T, et al. Extracellular vesicles including exosomes regulate innate immune responses to hepatitis B virus infection. Front Immunol 2016;7:335. https://doi.org/10.3389/fimmu.2016.00335.
[50] Wu Q, Glitscher M, Tonnemacher S, et al. Presence of intact hepatitis B virions in exosomes. Cell Mol Gastroenterol Hepatol 2022. https://doi.org/10.1016/j.jcmgh.2022.09.012. 0.
[51] Yang Y, Han Q, Hou Z, et al. Exosomes mediate hepatitis B virus (HBV) transmission and NK-cell dysfunction. Cell Mol Immunol 2017;14:465–475. https://doi.org/10.1038/cmi.2016.24.
[52] Kakizaki M, Yamamoto Y, Yabuta S, et al. The immunological function of extracellular vesicles in hepatitis B virus-infected hepatocytes. PLOS ONE 2018;13:e0205886. https://doi.org/10.1371/journal.pone.0205886.
[53] Klymiuk MC, Balz N, Elashry MI, et al. Exosomes isolation and identification from equine mesenchymal stem cells. BMC Vet Res 2019;15:42. https://doi.org/10.1186/s12917-019-1789-9.
[54] Pasloske BL, Walkerpeach CR, Obermoeller, et al. Armored RNA technology for production of ribonuclease-resistant viral RNA controls and standards. J Clin Microbiol 1998;36:3590–3594. https://doi.org/10.1128/JCM.36.12.3590-3594.1998.

Keywords: hepatitis B virus (HBV); chronic hepatitis B (CHB); biomarker; pregenomic RNA (pgRNA); standard.
Received 19 July 2023; received in revised form 16 May 2024; accepted 23 May 2024; Available online 25 May 2024

Journal of Hepatology, Volume *6*

Supplemental information

A molecular standard for circulating HBV RNA detection and quantification assays in patients with chronic hepatitis B

Alexia Paturel, Francesca Casuscelli di Tocco, Delphine Bousquet, Marie-Laure Plissonnier, Xavier Grand, Hyosun Tak, Françoise Berby, Caroline Scholtès, Barbara Testoni, Fabien Zoulim, and Massimo Levrero

A molecular standard for circulating HBV RNA detection and quantification assays in chronic hepatitis B patients

Alexia Paturel, Francesca Casuscelli di Tocco, Delphine Bousquet, Marie-Laure Plissonnier, Xavier Grand, Hyosun Tak, Françoise Berby, Caroline Scholtès, Barbara Testoni, Fabien Zoulim, Massimo Levrero

Table of contents

Fig. S1 2

Fig. S2 3

Fig. S3 4

Fig. S4 5

Fig. S5 6

Fig. S6 7

Figure S1

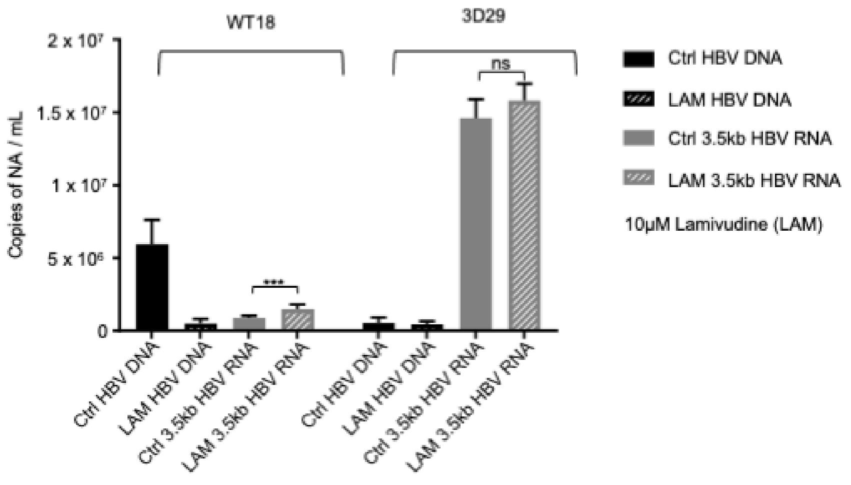

Fig. S1 – Effect of lamivudine treatment on viral nucleic acids secretion from the Huh7-WT18 and Huh7-3D29 cell clones. ddPCR quantification of HBV DNA and RNA secreted from Huh7-WT18 and Huh7-3D29 clonal cell lines untreated (Ctrl) or treated with 10µM of lamivudine (LAM). Huh7-WT18 control HBV RNA copies/mL in the supernatant vs Huh7-WT18 LAM treated cells (Paired t-test; P = 0.0001). Huh7-3D29 control HBV RNA copies/mL in the supernatant vs Huh7-3D29 LAM treated cells (Paired t-test; P = 0.3255). Validation of samples distribution as parametric by Shapiro-Wilk test. (Shapiro-Wilk p-value = 0.00062610). Error bars represent standard deviation of 2 technical replicates from each of 3 distinct cell passages, which served as biological replicates.

Figure S2

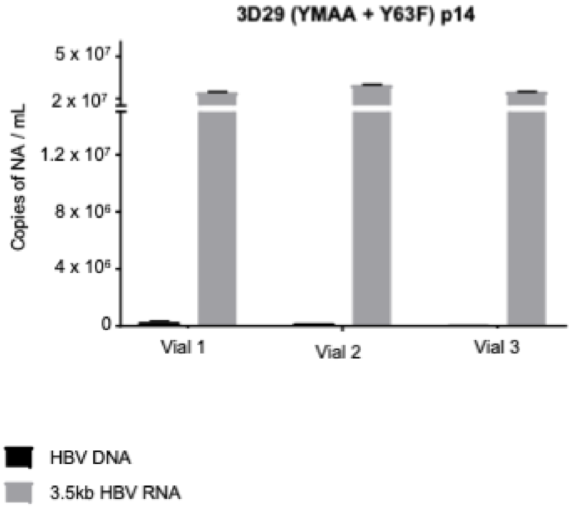

Fig. S2 - The Huh7-3D29 clone secretory phenotype is consistant between vials issued of the same culture passage. ddPCR quantification of secreted HBV DNA and RNA from Huh7-3D29 clone in different vials from passage 14 (one-way Kruskal-Wallis test, P = 0.0667).

Figure S3

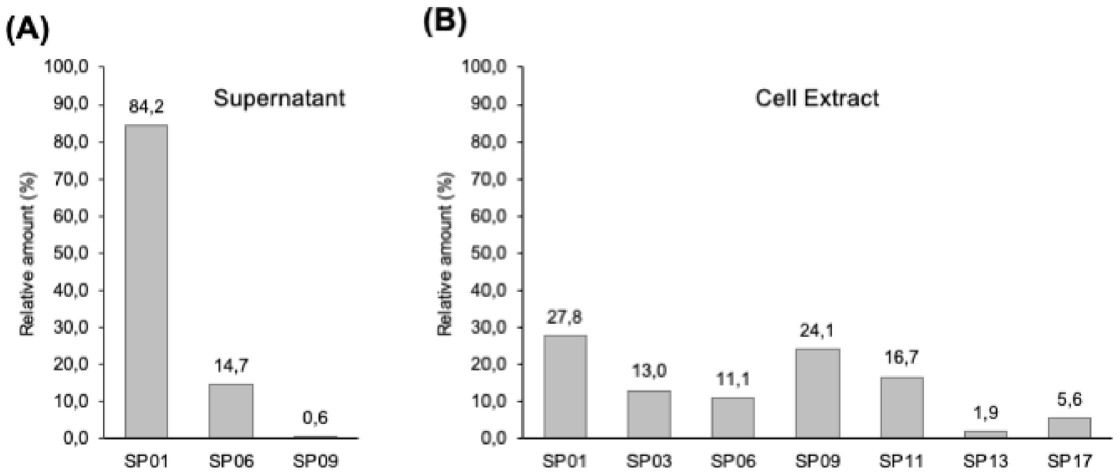

Fig. S3 - Percentage of each splice variant species of the 3.5kb RNA transcript. Values obtained from Nanopore sequencing performed on amplification products in the cell culture supernatants (*left panel*) and cellular extract of Huh7-3D29 cells (*right panel*) obtained after 5'RACE and calculated from transcripts identified as 3.5 according to TSS. All spliced variants below 0,3% was not included in the graph. SPs = splice variants.

Figure S4

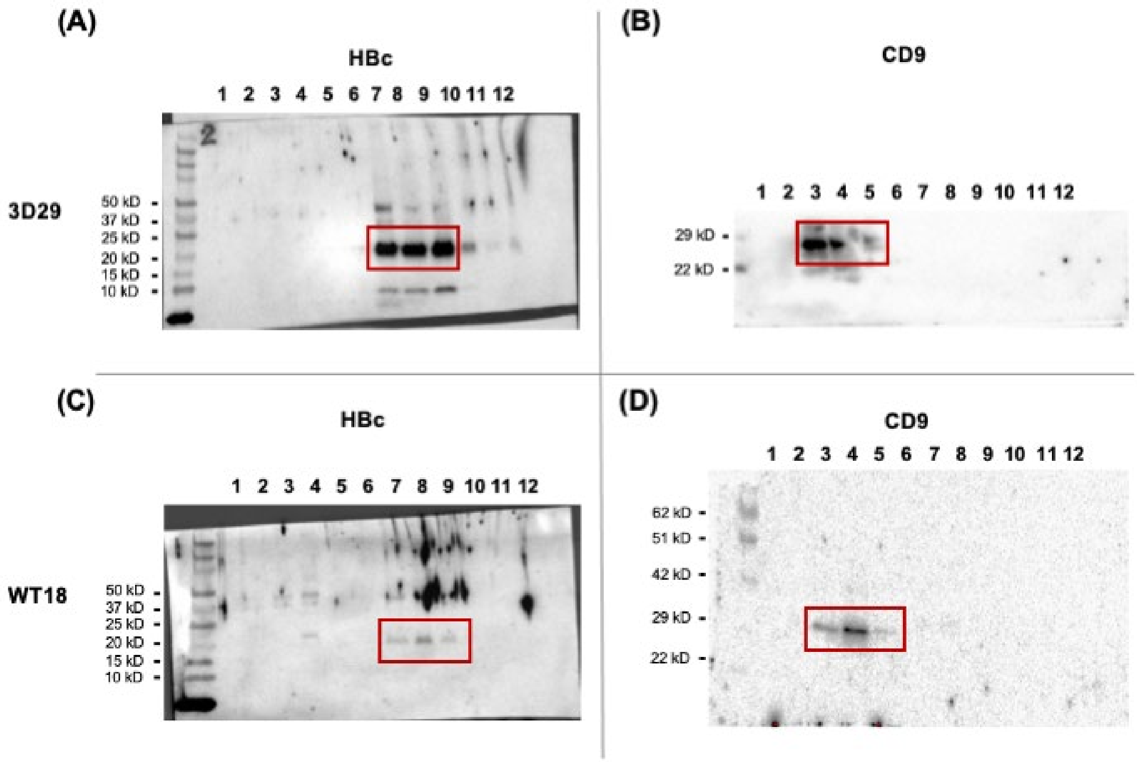

Figure S4 – Western Blot Raw data

Fig. S4 – HBc and CD9 immunoblot raw data. The entire immunoblots for the HBV capsid (HBc) and the sEV CD9 protein used to populate Figure 3 are shown. The figures on the top represents the 12 fractions obtained from the sucrose / iodixanol gradients of Huh7-3D29 (panels A and B) and Huh7-WT18 (panels C and D) cell supernatants. For the anti-HBc immunoblots (panels A and C) the Precision Plus Protein™ Prestained Protein Standard- (Biorad) was used. For the anti-CD9 immunoblots (panels B and D) the Opti-protein Marker (Applied Biological Material) and a Tris-Glycine 15% gel were used. In (B) the upper part of immunoblot membrane was cutted out in order to reduce the background noise and allow a longer exposure time.

Figure S5

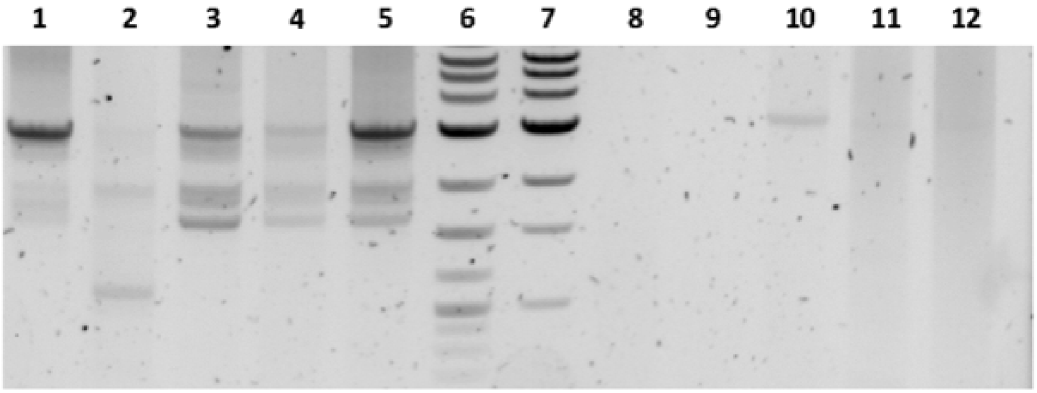

Fig. S5. PAGE analysis of the 5'RACE performed on cell extracts and culture supernatants. Lanes 3, 6 and 10 are included in the main figures as Figure 4A. Lanes 1-5: intracellular fractions (1 = HepAD38; 2 = PLC/PRF/5; 3 = Huh7-3D29; 4 = Huh7-5D1; 5 = Huh7-WT18). Lanes 6-7: molecular weight (MW) markers. Lanes 7-12: cell culture supernatants (8 = HepAD38; 9 = PLC/PRF/5; 10 = Huh7-3D29; 11 = Huh7-5D1; 12 = Huh7-WT18

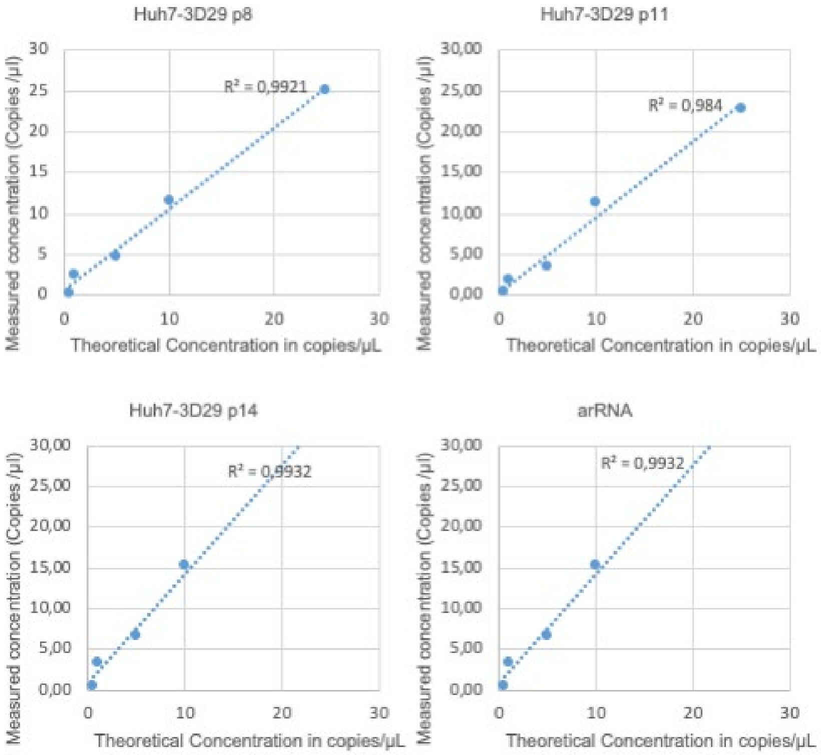

Fig. S6 – Performance of low concentration serial dilutions of Huh7-3D29 cell culture supernatant as a standard for HBV RNAs quantification assays. Serial dilutions from 25 until 0,5 copies/µl of Huh7-3D29 cell supernatants from 3 passages and of the HBV arRNA were used for the 2-step ddPCR. R2 = Spearman's coefficient correlation.
